# A simple, cost-effective high-throughput image analysis pipeline improves genomic prediction accuracy for days to maturity in wheat

**DOI:** 10.1186/s13007-020-00686-2

**Published:** 2020-11-02

**Authors:** Morteza Shabannejad, Mohammad-Reza Bihamta, Eslam Majidi-Hervan, Hadi Alipour, Asa Ebrahimi

**Affiliations:** 1 Department of Plant Breeding and Biotechnology, Faculty of Agricultural Sciences and Food Industries, Science and Research Branch, Islamic Azad University, P.O. Box 14515/775, Tehran, Iran; 2 Department of Agronomy and Plant Breeding, Faculty of Agricultural Sciences and Engineering, College of Agriculture and Natural Resources, University of Tehran, P.O. Box 4111, Karaj, Alborz Iran; 3 Department of Plant Production and Genetics, Faculty of Agriculture and Natural Resources, Urmia University, P.O. Box 165, Urmia, Iran

**Keywords:** High-throughput phenotyping, Image analysis, Pipeline, Genomic prediction, Days to maturity, Wheat

## Abstract

**Background:**

High-throughput phenotyping and genomic selection accelerate genetic gain in breeding programs by advances in phenotyping and genotyping methods. This study developed a simple, cost-effective high-throughput image analysis pipeline to quantify digital images taken in a panel of 286 Iran bread wheat accessions under terminal drought stress and well-watered conditions. The color proportion of green to yellow (tolerance ratio) and the color proportion of yellow to green (stress ratio) was assessed for each canopy using the pipeline. The estimated tolerance and stress ratios were used as covariates in the genomic prediction models to evaluate the effect of change in canopy color on the improvement of the genomic prediction accuracy of different agronomic traits in wheat.

**Results:**

The reliability of the high-throughput image analysis pipeline was proved by three to four times of improvement in the accuracy of genomic predictions for days to maturity with the use of tolerance and stress ratios as covariates in the univariate genomic selection models. The higher prediction accuracies were attained for days to maturity when both tolerance and stress ratios were used as fixed effects in the univariate models. The results of this study indicated that the Bayesian ridge regression and ridge regression-best linear unbiased prediction methods were superior to other genomic prediction methods which were used in this study under terminal drought stress and well-watered conditions, respectively.

**Conclusions:**

This study provided a robust, quick, and cost-effective machine learning-enabled image-phenotyping pipeline to improve the genomic prediction accuracy for days to maturity in wheat. The results encouraged the integration of phenomics and genomics in breeding programs.

## Background

The efficient and precise phenotyping of a large population is one of the main tasks in breeding programs [1]. For example, the recording process of grain yield is currently difficult, time-consuming, and costly. The visual assessments are normally incapable of attaining small but important phenotypic variations [2]. Even with good scoring, only small fractions of phenotypes like canopy color can be recorded with the use of visual assessments. The scoring methods cannot statistically indicate the effect of stress on diverse germplasms [1, 2].

Such barriers in phenotyping have motivated plant breeders to collaborate with engineers and invent modern technologies for high-throughput phenotyping (HTP) in greenhouses and fields [1]. The HTP will become more advantageous when it is a non-invasive and non-destructive method like proximal, remote sensing, and digital imaging [3]. The advances in data analysis have enabled machine learning (ML) to provide an accurate value of stress-related phenotypes [1]. A pipeline with a complete framework for fast feature extraction from high-throughput imaging can be used as a platform for real-time phenotyping [4–10].

The HTP platforms can include instruments such as RGB (read, green, blue), multispectral and hyperspectral cameras, spectrometer, normalized difference vegetation index (NDVI) sensors, and light detection and ranging (LiDAR) technology [1, 3, 11, 12]. The RGB cameras are widely used in field phenotyping, especially for estimating canopy coverage [13–16]. In addition, the RGB imaging is used as an alternative to NDVI in some researches [14–17]. The assessment of senescence [18, 19], crops nitrogen content [20], soil water evaporation [16], early vigor [21], and physiological yellowing [11] are conducted by digital RGB image analysis. Physiological yellowing which shows plant senescence and occurs naturally with time is used as an indicator of maturity or the impact of abiotic stress [11, 22]. Moreover, some researches have provided successful protocols for designing, developing, and deploying high-efficiency image analysis pipelines to assess the quantity of plant response to biotic and abiotic stresses [2, 23, 24]. High-throughput image analysis by computer vision and ML for phenotyping iron deficiency chlorosis (IDC) in soybean [1], hyperspectral imaging for drought stress in cereals [25], and thermal imaging in spinach [26] are some of the recent successful reports.

Drought stress in the Middle East usually occurs at the end of the growing season when spike has already appeared and seed is at the development stage. In the Persian plateau, where most of the environments are arid or semi-arid, farmers are well-trained over the centuries to store rainwater throughout spring and irrigate farms with the stored water at the end of the growing season. The Persian farmers irrigate their farms two to four more times with the stored water after spike appearance to avoid yield loss due to late-season drought stress. This strategy leads to a significant increase in wheat grain yield [27]. The impact of drought stress and irrigation at the end of the growing season on different genotypes needs further investigations.

Genomic prediction (GP) [28] methods use all genomic information irrespective of their position, status [quantitative trait locus (QTL), causal mutation, linked marker, etc.], and the specific effect on the trait of interest. The GP model trained in the training set (TS) will be applied to the validation set (VS) to estimate the accuracy of predictions. HTP and genomic selection (GS) accelerate genetic gain in breeding programs [3]. The use of a major QTL as a fixed effect in a GP model increases the accuracy of GP [1]. In wheat, the selection is accelerated by adding traits like canopy temperature (CT) and NDVI as secondary traits or covariates in GP models [3, 23, 29, 30].

Motivated by this, this study reported the impact of terminal drought stress (TDS) and well-watered (WW) conditions on days to maturity (DTM) in a highly diverse bread wheat germplasm through an ML-based image-phenotyping pipeline.

## Methods

### Plant materials and field trials

The association panel used in this study included 286 bread wheat accessions from Iran historical germplasm (199 landraces identified during 1931–1968 in the Persian plateau and 87 cultivars released during 1942–2014 in Iran). The plant materials were kindly provided by the University of Tehran (UT) and Seed and Plant Improvement Institute (SPII), Karaj, Iran. The detailed information about the association panel is provided in Additional file 1: Tables S1 and S2. The experiments were carried out at the Kheirabad Agricultural Research Station (36°31′51.7″N and 48°45′29.9″E) in Zanjan province during the 2017–2018 cropping season using two separate alpha lattice designs [31] with two replications for each. The plots were 1 m in length, 1 m in width, and 0.5 m apart. Drip irrigation method was used for watering with the use of two tapes for each plot. Irrigation was conducted every ten days until the spike appearance. Then, TDS was inducted by terminating irrigation for one design whereas another design WW for three more times.

### Genotyping and quality control

We used the genotyping-by-sequencing (GBS) [32] method for genetic fingerprinting and Poland et al. [33] method for library construction. The genotyping method has been described for the association panel, previously [34, 35]. Briefly, DNA was extracted by a modified cetyltrimethylammonium bromide (CTAB) method [36] and double-digested with *PstI* and *MspI* restriction enzymes, barcoded adapters were ligated to each DNA sample using T4 ligase, polymerase chain reactions (PCRs) were done using primers complementary to both adaptors, size-selection for 250–300 bp fragments was conducted using an E-gel system (Life Technologies, Inc.), and the size-selected library was sequenced on an Ion Proton sequencer (Life Technologies, Inc.). Sequence reads were trimmed to 64 bp sequences, and identical reads were grouped. Then, unique sequence tags were assigned to the sequence groups. The unique tags were aligned internally, whereas up to 3 bp interval alignment mismatch was allowed. The Trait Analysis by aSSociation Evolution and Linkage (TASSEL) software [37] was used to utilize the Universal Network Enabled Analysis Kit (UNEAK) pipeline [38] for SNP calling. SNPs with a missing rate of more than 20% and SNPs with a minor allele frequency (MAF) of less than 5% were removed. Unanchored SNPs were excluded too. The remaining missing data of the whole SNP data set were imputed in one step using the LD KNNi method [39] in the TASSEL software [37], whereas *K* *=* 10 was used in LD KNNi. Finally, 9047 SNPs were used for further analysis.

### Population structure and molecular markers estimates

The population structure was evaluated by the Bayesian clustering approach with the use of an admixture model in the STRUCTURE software [40]. The number of subpopulations (K) was assessed with the use of 10,000 burn-in and 10,000 Markov Chain Monte Carlo (MCMC) for K = 1–10 in 10 independent runs. The best K value was estimated by ΔK statistic [41] in the structure harvester website (https://taylor0.biology.ucla.edu/structureHarvester). Two subpopulations (SBP-I and -II) were identified within the association panel. The SNP calling was performed for each subpopulation and 7714 SNPs for SBP-I and 5873 SNPs for SBP-II were identified. A number of 4785 markers were common between SBP-I and SBP-II, which were systematically separated and named as common markers marker set (CMMS). The molecular markers estimates were assessed for each chromosome using the full matrix option in TASSEL software [37].

### Phenotypes

Phenotypic measurements included days to heading (DTH), days to maturity (DTM), duration of heading-to-maturity (DHTM), plant height (PH), and grain yield/m^2^ (GY). For details on measurements of DTH, DTM, DHTM, PH, and GY and time of assessments, please refer to the manual “Physiological breeding II: a field guide to wheat phenotyping” [42].

### Image acquisition

**Fig. 1:**
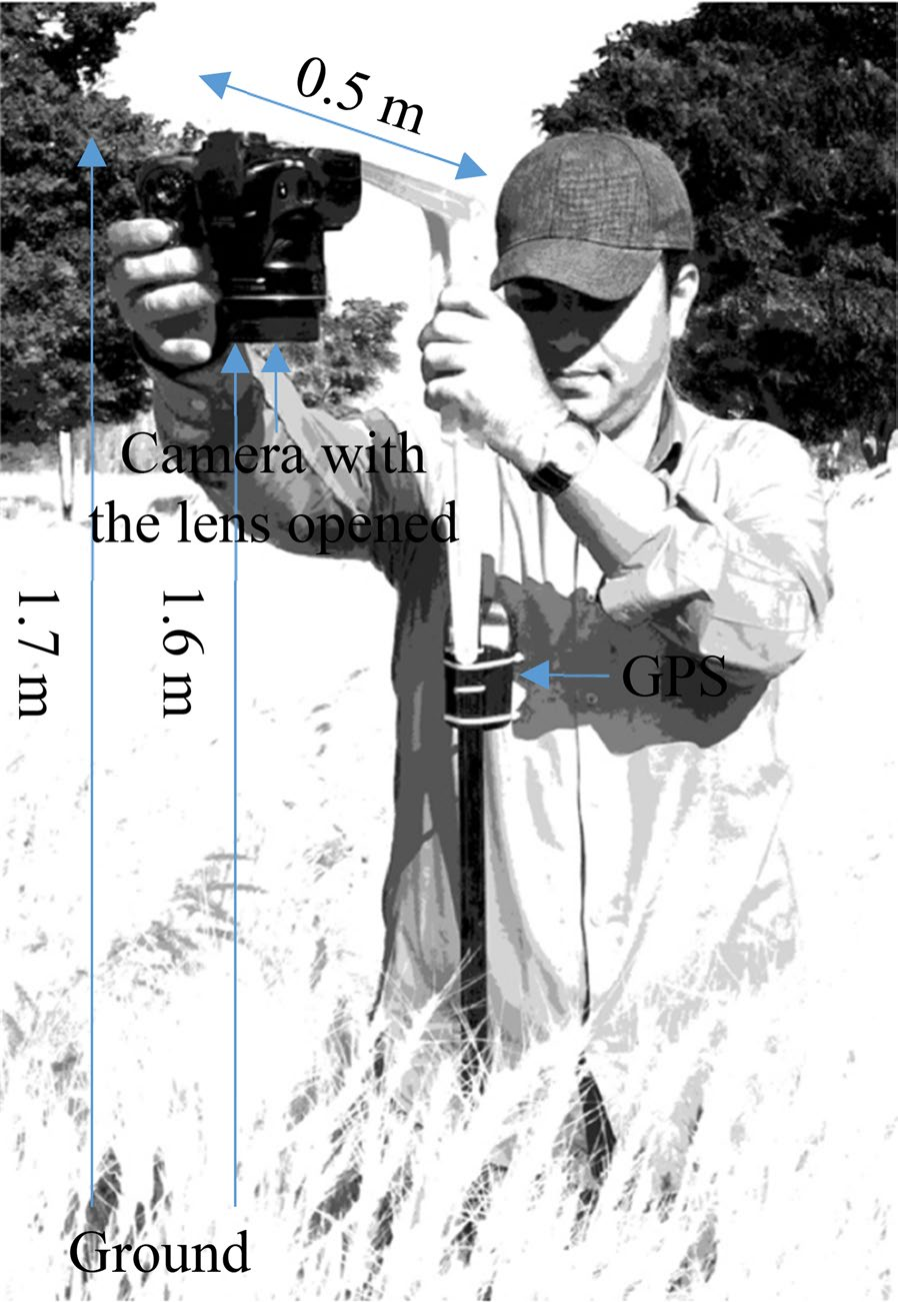
The imaging system. A Canon PowerShot SX30 IS camera and a global positioning system (GPS) were installed on a simple handheld phenocart. The phenocart height was 1.7 m. A flat L-shaped metal bar with 0.5 m long was installed on the phenocart. The camera was mounted on the L-shaped metal bar upside down, whereas the camera with the lens opened had 1.6 m distance from the ground. The GPS data are not used in the present study

A Canon PowerShot SX30 IS camera was installed on a simple handheld phenocart. The phenocart height was 1.7 m. A flat L-shaped metal bar with 0.5 m long was installed on the phenocart. The camera was mounted on the L-shaped metal bar upside down, whereas the camera with the lens opened had 1.6 m distance from the ground. The phenocart was on the right side of the plots during the imagings (Fig. 1).

The images were captured two weeks after TDS induction from the plots. In addition, the images were taken with the Scene Intelligent Auto mode of the camera during two consecutive days from 10 a.m. to 2 p.m. when the weather was completely sunny. Therefore, no color correction was applied to the captured images. The flash function was kept off to have stable light too. All of the images were taken as RGB and stored in JPEG format with a resolution of 4320 $\times$ 3240 pixels (Additional file 2: Figure S1). In total, 1144 images were taken two weeks after TDS induction and used in the ML model.

### Image processing

**Fig. 2:**
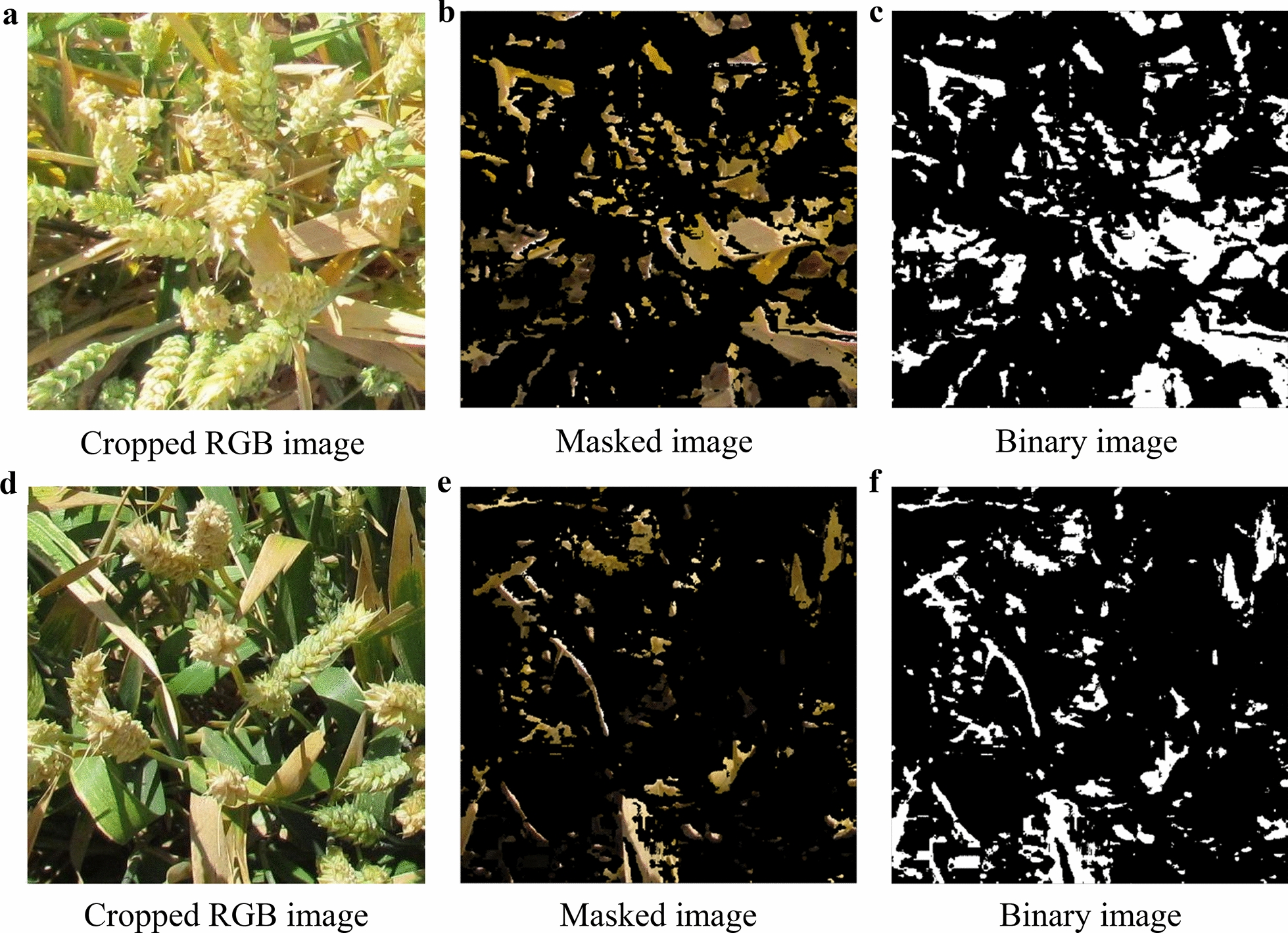
Image processing overview to assess tolerance and stress ratios under terminal drought stress (TDS) and well-watered (WW) conditions in wheat. **a** and **d** are cropped RGB images taken two weeks after drought stress induction under TDS and WW conditions, respectively. **b** and **e** are masked images in the Lab color space using defined function under TDS and WW conditions, respectively. **c** and **f** are masked images converted to binary format under TDS and WW conditions, respectively. Using this strategy, the black pixels represent non-dry tissues and the white pixels indicate dried tissues. The tolerance ratios were estimated as $Tolerance \,ratio\, \left( {TOR} \right) = \left( {Black \,pixels} \right)/\left( {White \, pixels} \right)$ and the stress ratios as $Stress\, ratio \, \left( {STR} \right) = \left( {White\, pixels} \right)/\left( {Black\, pixels} \right)$

In order to avoid shade, shoe, empty space, margin, etc. all of the images were cropped to 500 $\times$ 500 pixels using Preview software, so that the cropped images could represent the color of the canopies more precisely (Fig. 2a, d). A function was defined for the color threshold based on the CIELAB color space (L*a*b) [43] in MATLAB_R2015b software. The cropped RGB images were converted to L*a*b color space. The first channel (L, from black (0) to white ($+$ 100)) was kept intact, the second channel (a, from green ($-$ 100) to red ($+$ 100)) was converted to half and defined from 0 to $+$ 100, and the third channel (b, from blue ($-$ 100) to yellow ($+$ 100)) was also converted to half and defined from 0 to $+$ 100 (Fig. 2b, e). The masked images were converted to binary format (Fig. 2c, f). With the use of this strategy, the black pixels were an indicator of the range of cold colors (from the light illumination to the dark green and blue), and the white pixels were an indicator of the range of warm colors (from the light illumination to the dark red and yellow). Finally, the color proportion of the black to white pixels as a sign of the tolerance ratio (TOR) and the color proportion of the white to black pixels as a sign of the stress ratio (STR) were calculated for each plot and saved in a text file. The defined MATLAB function and the written code are provided in Additional file 3: Scripts S1 and S2.

### Data analysis

Analysis of variance (ANOVA) was carried out for each phenotype under TDS and WW conditions separately using the *proc mixed* procedure in SAS software version 9.4 [44]. The data analysis model was as follow:

$${y}_{ijk}=\mu +{g}_{i}+{r}_{j}+{b}_{k(j)}+{\varepsilon }_{ijk}$$

where ${y}_{ijk}$ represents the observed phenotype of the *i*th genotype at the *j*th replication of the *k*th block within the *j*th replication, $\mu$ represents the overall mean, ${g}_{i}$ indicates the genetic effect of the *i*th genotype, ${r}_{j}$ indicates the effect of the *j*th replication, ${b}_{k(j)}$ shows the *k*th block effect within the *j*th replication and ${\varepsilon }_{ijk}$ shows the residual effect following $N(0, {\sigma }_{\varepsilon }^{2}$). All effects were considered as random. The estimation of variance components was performed by the *proc varcomp* procedure, whereas all effects were considered as random. Heritability (${H}^{2}$) estimates were calculated based on each accession mean with an assumption of independence of effects using the following equation:

$${H}^{2}={\sigma }_{g}^{2}/{(\sigma }_{g}^{2}+{\sigma }_{\varepsilon }^{2}/r)$$

where ${\sigma }_{g}^{2}$, ${\sigma }_{\varepsilon }^{2}$, and $r$ represent the genotypic variance, residual variance, and the number of replications, respectively [45]. Best linear unbiased predictions (BLUPs) of genetic effect for each genotype were estimated under TDS and WW conditions using the R package lme4 [46] in the same model as described for the phenotypic analyzes. Then, the BLUPs were used for GP assessments.

### GP strategy

For five-fold cross-validation (CV), 20% of accessions were randomly assigned to a VS, whereas all of the remaining genotypes were used as a TS. The whole process was repeated 100 times for each GP (The Bayesian analyses were implemented along with 10,000 iterations and 1000 burn-ins). The CMMS was used as a marker set for assessing genomic estimated breeding values (GEBVs). The accuracy of the GP was estimated as Pearson’s correlation coefficient among GEBVs and BLUPs over TS and VS. The average of accuracies was reported across folds and repeats [47]. The GPs were implemented with seven different methods including genomic best linear unbiased prediction (GBLUP), ridge regression-best linear unbiased prediction (RR-BLUP), Bayesian A (BA), Bayesian B (BB), Bayesian C $\pi$ (BC $\pi$), Bayesian LASSO (BL), and Bayesian ridge regression (BRR) in iPat software [48]. A brief review of the GP methods is provided by Juliana et al. [49].

Four univariate (UV) GP models were defined. Five phenotypes (DTH, DTM, DHTM, PH, and GY) were evaluated in each of the UV models under TDS and WW conditions, separately. The UV1 model did not contain any covariate. TOR as a covariate was included in the UV2 model. STR as a fixed effect was included in the UV3 model. Both TOR and STR as fixed effects were included in the UV4 model.

In total, 280 analyses were conducted including 4 UV models, 5 phenotypes, 2 irrigation conditions, and 7 GP methods.

## Results

### Field conditions

Plantings were conducted at the Kheirabad Agricultural Research Station in Zanjan province in the middle of October and weather conditions were recorded during the cropping season (Additional file 4: Figure S2). Zanjan province is located in a cold semi-arid climate zone.

### Population structure and distribution of molecular markers

The existence of two main subpopulations was identified using the ΔK statistic (Additional file 5: Figure S3). The cluster membership coefficients (Q) indicated that the SBP-I contained 77 cultivars and 71 landraces, and the SBP-II included 128 landraces and ten cultivars (Additional file 6: Table S3). In the whole association panel, the highest number of markers was on chromosome 2B (419), while the lowest number of SNPs was on chromosome 4D (34) (Table 1). The genetic map length was the longest for chromosome 3A (171.063 cM), while the shortest length was for chromosome 2D (85.027 cM) (Table 1). The highest marker density was on chromosome 2B (3.76 Marker/cM), while the lowest marker density was on chromosome 4D (0.38 Marker/cM) (Table 1). The B genome had the highest number of markers (2197), followed by the A (1794) genome and the D genome (794) (Table 1).

**Table 1:** Distribution of molecular markers in an association panel including 286 Iran bread wheat accessions

Genome	Chromosome	Molecular markers distribution		
		Number of markers	Genetic map length (cM)	Marker density (marker/cM)
A genome	1A	261	117.878	2.21
	2A	314	92.517	3.39
	3A	227	171.063	1.33
	4A	195	152.121	1.28
	5A	190	111.967	1.70
	6A	246	99.391	2.48
	7A	361	135.625	2.66
	Total	1794	880.562	2.04
B genome	1B	299	113.814	2.63
	2B	419	111.506	3.76
	3B	364	121.909	2.99
	4B	110	102.696	1.07
	5B	349	155.004	2.25
	6B	301	97.872	3.08
	7B	355	118.551	2.99
	Total	2197	821.352	2.67
D genome	1D	166	123.978	1.34
	2D	140	85.027	1.65
	3D	97	126.448	0.77
	4D	34	90.119	0.38
	5D	74	170.702	0.43
	6D	121	121.074	1.00
	7D	162	157.445	1.03
	Total	794	874.793	0.91
Whole genome		4785	2576.707	1.85

### Phenotypic data summary

The descriptive statistics, variance parameters (${\sigma }_{G}^{2}$ and ${\sigma }_{E}^{2}$), and heritability (${H}^{2}$) were estimated for all traits under TDS and WW conditions, separately (Table 2). All traits had higher phenotypic values under the WW conditions (except STR) compared to the TDS conditions (Table 2). In addition, the higher estimates of ${\sigma }_{G}^{2}$, ${\sigma }_{E}^{2}$, and ${H}^{2}$ were observed for all traits (except STR) under the WW conditions (Table 2). Pearson correlation coefficients were calculated for all traits under both TDS and WW conditions (Table 3). The DTH and DHTM indicated the highest correlations under TDS and WW conditions (− 0.68 and − 0.73, respectively) (Table 3). Furthermore, the DTH and PH were correlated under TDS and WW conditions (0.57 and 0.60, respectively) (Table 3). The DTM and DHTM were positively (0.58 and 0.44, respectively) correlated under TDS and WW conditions (Table 3). However, the DHTM and PH were negatively (− 0.35 and − 0.40, respectively) correlated under TDS and WW conditions (Table 3). The GY correlation with DHTM was low under TDS and WW conditions (0.19 and 0.20, respectively) (Table 3). TOR had positive correlation with DTM (0.32) under TDS conditions, and with DTH (0.19), DTM (0.28), and PH (0.19) under WW conditions (Table 3). STR demonstrated negative correlations with DTM (− 0.36 and − 0.26) and TOR (− 0.46 and − 0.27) under TDS and WW conditions, respectively (Table 3).

**Table 2:** Descriptive statistics and variance parameters for seven traits in an association panel including 286 Iran breed wheat accessions grown under terminal drought stress (TDS) and well-watered (WW) conditions in semi-arid environments, Iran

Condition	Trait	Descriptive statistics			Variance parameters		
		Min	Mean	Max	${\sigma }_{G}^{2}$	${\sigma }_{E}^{2}$	${H}^{2}$
TDS	DTH	196	217.22	230	29.01	8.35	0.87
	DTM	251	264.37	276	10.38	8.58	0.71
	DHTM	27	47.17	68	2.10	2.04	0.67
	PH	65	114.28	151	18.33	10.14	0.78
	GY	0.16	0.29	0.59	0.15	0.14	0.68
	TOR	0.13	3.55	30.81	5.36	11.97	0.47
	STR	0.03	0.72	7.82	0.41	0.26	0.76
WW	DTH	200	217.26	230	30.91	8.64	0.88
	DTM	256	270.96	280	15.27	9.40	0.76
	DHTM	36	53.71	71	3.58	2.85	0.72
	PH	68	118.36	164	26.35	11.29	0.82
	GY	0.19	0.37	0.71	0.18	0.15	0.71
	TOR	0.36	33.16	503.03	606.33	1900.4	0.39
	STR	0.00	0.13	2.77	0.01	0.05	0.29

${\sigma }_{G}^{2}$ genotype variance, ${\sigma }_{E}^{2}$ residual variance, ${H}^{2}$ heritability, *DTH* days to heading, *DTM* days to maturity, *DHTM* duration of heading-to-maturity, *PH* plant height (cm), *GY* grain yield (kg/m^2^), *TOR* tolerance ratio, *STR* stress ratio

**Table 3:** Pearson correlation coefficients for seven traits in an association panel including 286 Iran bread wheat accessions grown under terminal drought stress (TDS) and well-watered (WW) conditions in semi-arid environments, Iran

Trait	DTH	DTM	DHTM	PH	GY	TOR	STR
DTH		0.30**	− 0.73**	0.60**	− 0.11*	0.19**	− 0.02^ ns^
DTM	0.21**		0.44**	0.23**	0.14**	0.28**	− 0.26**
DHTM	− 0.68**	0.58**		− 0.40**	0.20**	0.03^ ns^	− 0.17**
PH	0.57**	0.17**	− 0.35**		− 0.12*	0.19**	− 0.02^ ns^
GY	− 0.10*	0.14**	0.19**	− 0.11*		0.02^ ns^	− 0.16**
TOR	0.12**	0.32**	0.14**	0.09*	0.12**		− 0.27**
STR	− 0.12**	− 0.36**	− 0.17**	− 0.13**	− 0.10*	− 0.46**	

The numbers under the diagonal indicate correlation under TDS conditions. The numbers above the diagonal indicate correlation under WW conditions

*DTH* days to heading, *DTM* days to maturity, *DHTM* duration of heading-to-maturity, *PH* plant height (cm), *GY* grain yield (kg/m^2^), *TOR* tolerance ratio, *STR* stress ratio

^ns^ non-significant

^*^P ≤ 0.05

^**^P ≤ 0.01

### GP

The prediction accuracies varied from − 0.06 to 0.45 (Table 4). None of the traits indicated high prediction accuracy in the UV1 model, where no fixed effect was utilized in the GP models to estimate GEBVs (Table 4). The prediction accuracy was increased for DTH (0.19), DTM (0.39), DHTM (0.16), PH (0.11) and GY (0.16) under TDS conditions and for DTH (0.23), DTM (0.37) and PH (0.23) under WW conditions using the UV2 model, where TOR was included as a covariate in the GP models (Table 4). Further, the prediction accuracy was improved for DTH (0.16), DTM (0.42), DHTM (0.21), PH (0.15) and GY (0.13) under TDS conditions and for DTM (0.36), DHTM (0.23) and GY (0.19) under WW conditions using the UV3 model, where STR served as a fixed effect in the GP models (Table 4). The prediction accuracy was higher for DTM (0.45) under TDS conditions and for DTM (0.42), DHTM (0.23), PH (0.25) and GY (0.19) under WW conditions using the UV4 model, where both TOR and STR were included as covariates in the GP models (Table 4). None of the highest of accuracies was identified using the BA and BB methods in the UV2, UV3, and UV4 models (Table 4). The prediction accuracies of the DTM were increased three to four times using the UV2, UV3, and UV4 models under both TDS and WW conditions (Table 4).

**Table 4:** Genomic prediction (GP) accuracy for five agronomic traits in an association panel including 286 Iran bread wheat accessions grown under terminal drought stress (TDS) and well-watered (WW) conditions using high-throughput image analysis results as fixed effects in the univariate (UV) GP models

Model	Method	TDS					WW				
		DTH	DTM	DHTM	PH	GY	DTH	DTM	DHTM	PH	GY
UV1	RR-BLUP	0.10	0.10	0.10	0.06	0.06	0.09	0.08	0.04	0.06	0.05
	GBLUP	0.12	0.10	0.10	0.06	0.07	0.09	0.08	0.04	0.06	0.06
	BA	0.11	0.11	0.10	− 0.05	0.11	0.09	0.09	0.02	− 0.04	0.12
	BB	0.11	0.11	0.10	− 0.02	0.10	0.10	0.09	0.03	− 0.02	0.12
	BC $\pi$	0.12	0.11	0.10	− 0.01	0.10	0.09	0.09	0.04	− 0.01	0.11
	BL	0.11	0.12	0.10	− 0.06	0.08	0.11	0.08	0.03	− 0.04	0.12
	BRR	0.11	0.10	0.10	− 0.03	0.10	0.10	0.09	0.03	− 0.01	0.11
UV2	RR-BLUP	0.15	0.38	0.16	0.11	0.15	0.23	0.37	0.01	0.23	− 0.03
	GBLUP	0.17	0.38	0.15	0.10	0.12	0.21	0.36	− 0.01	0.23	− 0.03
	BA	0.18	0.37	0.15	0.01	0.15	0.19	0.32	0.03	0.13	0.11
	BB	0.18	0.38	0.15	0.02	0.15	0.21	0.34	0.03	0.16	0.10
	BC $\pi$	0.18	0.38	0.16	0.03	0.16	0.22	0.34	0.03	0.18	0.09
	BL	0.19	0.37	0.15	− 0.02	0.14	0.19	0.34	0.02	0.10	0.07
	BRR	0.19	0.39	0.16	0.04	0.16	0.22	0.34	0.04	0.19	0.09
UV3	RR-BLUP	0.15	0.42	0.19	0.15	0.12	− 0.05	0.36	0.23	− 0.06	0.19
	GBLUP	0.14	0.42	0.19	0.13	0.09	0.01	0.35	0.22	− 0.02	0.17
	BA	0.15	0.39	0.20	0.02	0.12	0.09	0.31	0.17	− 0.04	0.18
	BB	0.15	0.41	0.20	0.05	0.12	0.08	0.31	0.18	− 0.02	0.18
	BC $\pi$	0.15	0.41	0.21	0.06	0.13	0.08	0.32	0.19	− 0.01	0.18
	BL	0.16	0.40	0.20	0.01	0.11	0.06	0.30	0.16	− 0.05	0.18
	BRR	0.16	0.41	0.21	0.07	0.13	0.08	0.32	0.20	− 0.01	0.19
UV4	RR-BLUP	0.14	0.44	0.17	0.14	0.13	0.22	0.42	0.23	0.25	0.18
	GBLUP	0.16	0.44	0.18	0.12	0.12	0.21	0.41	0.21	0.24	0.16
	BA	0.17	0.44	0.19	0.02	0.15	0.20	0.38	0.16	0.15	0.18
	BB	0.17	0.44	0.19	0.05	0.14	0.20	0.39	0.17	0.18	0.18
	BC $\pi$	0.17	0.44	0.20	0.06	0.14	0.21	0.40	0.19	0.20	0.17
	BL	0.17	0.43	0.20	− 0.01	0.16	0.20	0.38	0.13	0.11	0.19
	BRR	0.18	0.45	0.20	0.07	0.15	0.21	0.39	0.19	0.20	0.18

The average of accuracies was reported across folds and repeats. No covariate was used in the UV1 model. TOR as a covariate was used in the UV2 model. STR as a covariate was used in the UV3 model. Both TOR and STR as covariates were utilized in the UV4 model

*GBLUP* genomic best linear unbiased prediction, *RR-BLUP* ridge regression-best linear unbiased prediction, *BA* Bayesian A, *BB* Bayesian B, *BC *$\pi$ Bayesian C $\pi$, *BL* Bayesian LASSO, *BRR* Bayesian ridge regression, *DTH* days to heading, *DTM* days to maturity, *DHTM* duration of heading-to-maturity, *PH* plant height (cm), *GY* grain yield (kg/m^2^), *TOR* tolerance ratio, *STR* stress ratio

## Discussion

Phenotypes with stable heritability are less sensitive to the GP method [50, 51]. DTH, PH, and NDVI showed high heritability and were used as fixed effects in some studies [3, 23, 29, 45]. Heritability and correlation among traits are important factors to attain higher prediction accuracy [3]. High broad‐sense heritability (> 0.57) was observed for wheat vegetation indices with the use of unmanned aerial systems (UAS) [23]. In addition, the visual and digital assessments showed a 0.95 correlation for the physiological yellowing in wheat, whereas the digital assessments had 0.76 heritability [11]. In the present study, regardless of the correlation values, all agronomic traits had a positive correlation with TOR and a negative correlation with STR under both TDS and WW conditions, respectively. The heritability of TOR was 0.76 under TDS conditions, and the heritability of STR was 0.29 under WW conditions. As a conclusion, positive correlation and high heritability of TOR with DTM under TDS conditions, as well as negative correlation and low heritability of STR with DTM under WW conditions indicated the high adaptability of the association panel to drought stress.

In this study, the whole association panel was a mixed population (87 cultivars and 199 landraces). More accurate results were reported from mixed populations because more diversity in TS and more inbred genotype in VS would be available during the CVs [52–55]. In the breeding programs, a diverse or an inbred VS would be compared with a large and diverse TS containing high genetic diversity [53]. This approach will prevent the occurrence of a full relationship among genotypes in TS and VS, and consequently, more reliable results will be obtained [56, 57]. Higher marker density will provide better prediction accuracy [58, 59]. However, if MS covers the whole genome appropriately, the GP can predict all QTLs with stable linkage disequilibrium (LD) across subpopulations [28, 60, 61]. The present study used the markers which were common between subpopulations to obtain higher prediction accuracy [62]. RR-BLUP and GBLUP are mathematically equivalent [49]. RR-BLUP demonstrates more reliable results for QTLs with small effects [50]. If TS is closely related to the selected candidates, the GBLUP method will obtain a more nonadditive genetic variance [63]. The Bayesian methods can provide better results when the number of QTLs decreases and effect increases [51]. The genetic architecture of phenotypes would change GEBV [64, 65]. In addition, adding secondary traits or covariates to the UV and multivariate (MV) GP models would increase prediction accuracy [3, 29, 45]. The results of the present study showed that all of the GP methods had the highest prediction accuracy for DTM (0.38–0.45) when both TOR and STR were used in the UV4 model under both TDS and WW conditions.

The CT and NDVI as secondary traits in wheat improved the prediction accuracy of GY by 70% [29]. Furthermore, the manually taken images indicated 0.61–0.78 correlation with the visual scoring of the physiological yellowing in wheat [11]. The GP accuracy was about 0.30 using CT and NDVI as covariates in the UV models [45]. In this study, the prediction accuracies were increased to 0.39 and 0.42 for DTM using TOR and STR as separated covariates in the UV2 and UV3 models, under TDS conditions compared to the UV1 GP model. Further, the prediction accuracies increased for DTM to 0.37 and 0.36 using TOR and STR as separated covariates in the UV2 and UV3 models, under WW conditions compared to the UV1 GP model. A combination of TOR and STR as joint covariates in the UV4 model increased prediction accuracies for DTM to 0.45 and 0.42 under TDS and WW conditions, respectively. Therefore, the present study concluded that adding TOR and STR to the UV GP models can improve prediction accuracies. The above-mentioned results showed an improvement in the GP accuracy for DTM in a cost-effective way.

## Conclusions

The present study activated an ML-enabled image analysis pipeline to identify TOR and STR impact on the GP of the DTM under TDS and WW conditions. The results revealed the reliability of this pipeline for quantifying small phenotypic variations and integrating its advantages in genomic studies. The high prediction accuracy proves the benefit of utilizing TOR and STR as fixed effects in the UV GP models for DTM. The presented high-throughput image analysis pipeline can be generalized for evaluating other crops. In addition, the installation of this pipeline into aerial and ground-based systems promises to accelerate genetic gain in breeding programs.

## Supplementary information

**Additional file 1: Tables S1.** and **S2** are lists of the 199 landraces and 87 cultivars from Iran bread wheat germplasm used in the present study.

**Additional file 2: Figure S1.** demonstrates general conditions of a plot during the imaging.

**Additional file 3: Scripts S1.** and **S2** are defined function and written code in MATLAB.

**Additional file 4: Figure S2.** shows climate conditions in the field.

**Additional file 5: Figure S3.** demonstrates ∆K values for population structure.

**Additional file 6: Table S3.** provides information about the members of each subpopulation.

## Data Availability

The images and datasets used and analyzed during the present study are available from the corresponding author on reasonable request.

## Abbreviations

ANOVA: Analysis of variance
BA: Bayesian A
BB: Bayesian B
BC: Bayesian C
BL: Bayesian LASSO
BRR: Bayesian ridge regression
BLUPs: Best linear unbiased predictions
CT: Canopy temperature
CMMS: Common markers marker set
CV: Cross-validation
DTH: Days to heading
DTM: Days to maturity
DHTM: Duration of heading-to-maturity
GBLUP: Genomic best linear unbiased prediction
GBS: Genotyping-by-sequencing
GEBV: Genomic estimated breeding value
GP: Genomic prediction
GS: Genomic selection
GPS: Global positioning system
GY: Grain yield
HTP: High-throughput phenotyping
LiDAR: Light detection and ranging
LD: Linkage disequilibrium
ML: Machine learning
MCMC: Markov Chain Monte Carlo
MAF: Minor allele frequency
MV: Multivariate
NDVI: Normalized difference vegetation index
IDC: Phenotyping iron deficiency chlorosis
PCR: Polymerase chain reaction
PH: Plant height
QTL: Quantitative trait loci
RR-BLUP: Ridge regression-best linear unbiased prediction
SPII: Seed and Plant Improvement Institute
SNP: Single nucleotide polymorphism
STR: Stress ratio
SBP: Subpopulation
TDS: Terminal drought stress
TOR: Tolerance ratio
TS: Training set
TASSEL: Trait Analysis by aSSociation Evolution and Linkage
UV: Univariate
UNEAK: Universal Network Enabled Analysis Kit
UT: University of Tehran
UAS: Unmanned aerial systems
VS: Validation set
WW: Well-watered
SRBIAU: Science and research branch, Islamic Azad University
