# Supplementary figures and images for: A simple, cost-effective high-throughput image analysis pipeline improves genomic prediction accuracy for days to maturity in wheat
Source: Plant Methods. 2020 Nov 2;16:146. doi: 10.1186/s13007-020-00686-2 (PMC7607823; doi:10.1186/s13007-020-00686-2)

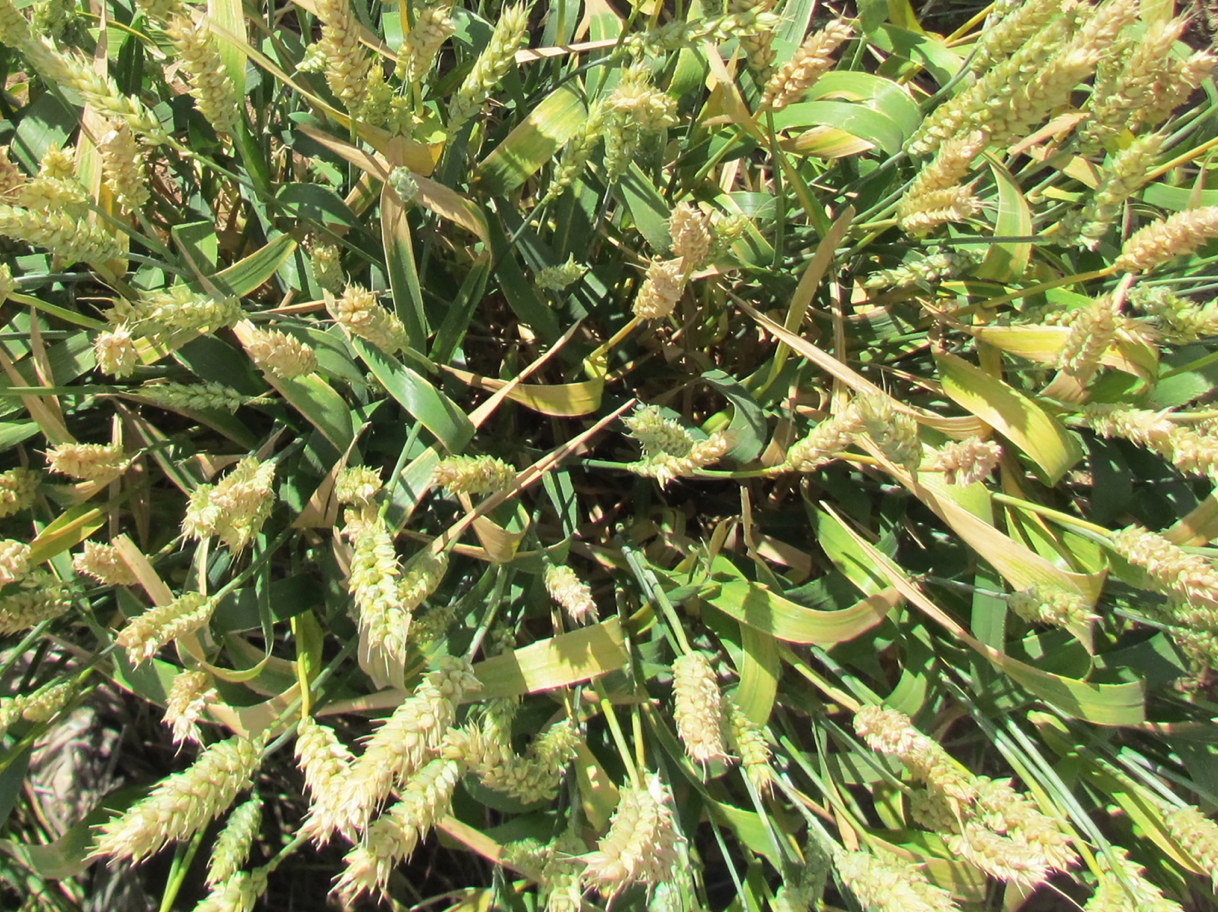


Fig. S1. The general conditions of a plot during the imaging.

Supplement: Supplementary file 2 — Additional file 2: Figure S1. demonstrates general conditions of a plot during the imaging. [file 13007_2020_686_MOESM2_ESM.docx]
