# Supplementary material for: A simple, cost-effective high-throughput image analysis pipeline improves genomic prediction accuracy for days to maturity in wheat
Source: Plant Methods. 2020 Nov 2;16:146. doi: 10.1186/s13007-020-00686-2 (PMC7607823; doi:10.1186/s13007-020-00686-2)
Supplement: Supplementary file 4 — Additional file 4: Figure S2. shows climate conditions in the field. [file 13007_2020_686_MOESM4_ESM.docx]

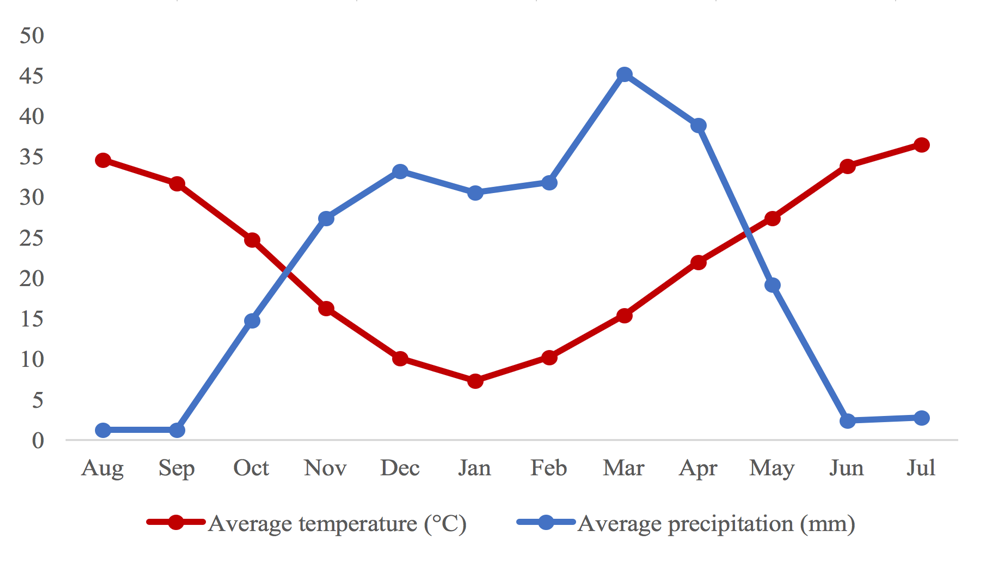


Fig. S2 Climate condition including average temperature (^o^C) and precipitation (mm) at the Kheirabad Agricultural Research Station in Zanjan province during the 2017-2018 cropping season.
