# Supplementary material for: A simple, cost-effective high-throughput image analysis pipeline improves genomic prediction accuracy for days to maturity in wheat
Source: Plant Methods. 2020 Nov 2;16:146. doi: 10.1186/s13007-020-00686-2 (PMC7607823; doi:10.1186/s13007-020-00686-2)
Supplement: Supplementary file 5 — Additional file 5: Figure S3. demonstrates ∆K values for population structure. [file 13007_2020_686_MOESM5_ESM.docx]

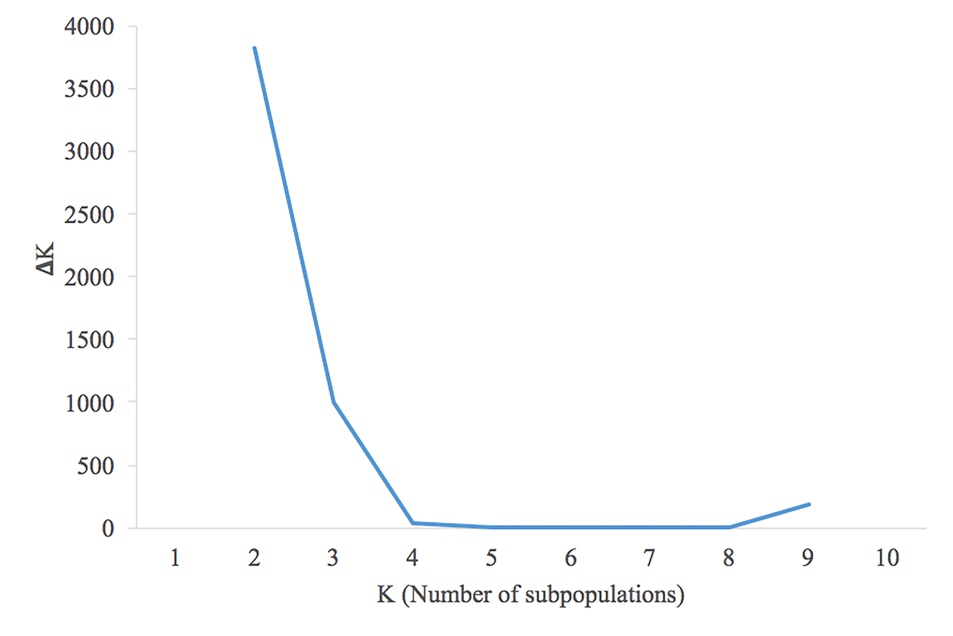


Fig. S3 ∆K values calculated for K = 1 to 10 to determine the number of subpopulations in a panel of 286 bread wheat accessions from Iran historical germplasm.
